# Reversed shoulder arthroplasty in cuff tear arthritis, fracture sequelae, and revision arthroplasty: Outcome in 59 patients followed for 2–7 years

**DOI:** 10.3109/17453674.2010.487242

**Published:** 2010-05-21

**Authors:** Annika Stechel, Uwe Fuhrmann, Lars Irlenbusch, Olaf Rott, Ulrich Irlenbusch

**Affiliations:** ^1^Department of Orthopaedic Surgery, Marienstift Arnstadt, Arnstadt; ^2^Department of Traumatology, Martin Luther University, HalleGermany

## Abstract

**Background and purpose:**

Reversed shoulder arthroplasty may be used for severe arthropathy where conventional prostheses cannot restore the function sufficiently. We analyzed the medium–term results and potential complications of the reversed prostheses, and also the influence of etiology on the result.

**Methods:**

52 women and 7 men, average age 70 (60–82) years, were followed for mean 4 (2–7) years. The indications were cuff tear arthropathy (CTA) (23), fracture sequelae (20), and revision of a failed conventional arthroplasty (16).

**Results:**

The average Constant score improved from 18 (2–55) points to 59 (17–96) points. It rose from 26 to 74 points in patients with CTA, from 12 to 48 in those with fracture sequelae, and from 10 to 54 points in revision arthroplasty. We also found an overall improvement in active forward flexion from 47° to 105°, and in active abduction from 46° to 93°. Scapular notching was seen in 51 shoulders. Radiolucent lines below the base–plate were present in 2 cases. There were no instances of loosening. Revisions were necessary in 15 patients: 5 with infections (all had had prior surgery), 5 with hematoma, 3 with dislocations, and 2 with disconnections of the shaft components.

**Interpretation:**

Reversed prosthetic replacement is a suitable method for restoring function and attaining pain relief in severe arthropathies. The results in revision arthroplasty are less predictable, with complications and revision rates higher than those in CTA patients. The reversed prosthesis should therefore only be used when conventional methods have failed.

## Introduction

A painful malfunctioning shoulder due to joint incongruence in combination with a loss of the centering action of the rotator muscles may be caused by severe rotator cuff arthritis, malunited/pseudarthrotic fractures, or loss of the tubercles in prosthesis revision. Contraction of the deltoid muscle when raising the arm induces a cranial migration of the head. An anatomically unconstrained prosthesis does not restore a stable center of rotation and is likely to fail (Nwakama et al. 2000, Edwards et al. 2002).

A reversed prosthesis is a way out of this dilemma, since it allows reconstruction of a stable center of rotation besides replacement of the articular surfaces (Grammont and Baulot 1993, Boileau et al. 2001, Sirveaux et al. 2004, Jouve et al. 2006, Molé et al. 2006, Wall et al. 2006, Bufquin et al. 2007, Irlenbusch et al. 2008c). Despite severe damage and loss of balance in the rotator cuff, free elevation can be attained through the sole action of the external shoulder muscles.

We compared the results and the complication rates after implantation of a reversed prosthesis in the 3 diagnosis groups. Preoperatively, the groups showed substantial differences in terms of loss of function and degree of destruction.

## Patients and methods

We treated 68 consecutive shoulders with a reversed Delta III prosthesis (DePuy) during the period 2002 – 2007. 59 patients (52 women) with an average age of 70 (60–82) years participated in this study and they were followed prospectively for mean 4 (2–7) years. The indications were cuff tear arthropathy (CTA) in 23 patients, fracture sequelae in 20 patients, and revision of a conventional prosthesis in 16 patients.

9 patients did not show up for follow–up because of living too far away (3) or because they were satisfied with the result and did not see any reason for a repeat examination (2). 1 patient was dissatisfied and had undergone further surgery, and 3 could not be reached.

### Clinical criteria

The clinical outcome was evaluated using the Constant score, by investigators not involved in the surgery. Strength was measured with a tensiometer at the wrist joint in maximal abduction in the scapular plane. Preoperative and postoperative mobility and pain relief were registered.

### Radiographic criteria

**Figure 1.**
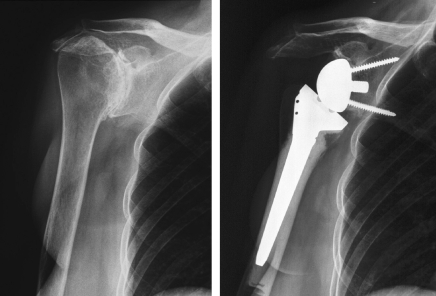
Delta III prosthesis in CTA type V according to Hamada.

CTA was classified according to Hamada et al. (1990). None of the 23 patients in the CTA group were assigned to group I, 2 patients were in group II, (incipient decentering, acromiohumeral distance/AHD ≤ 5 mm), 6 patients were in group III (decrease of the AHD and acetabularization), 11 patients were in group IV (additional narrowing of the articular space/osteoarthritis), and 4 patients were in group V (manifest cuff tear arthropathy with collapse of the head of the humerus) (Figure 1).

**Figure 2.**
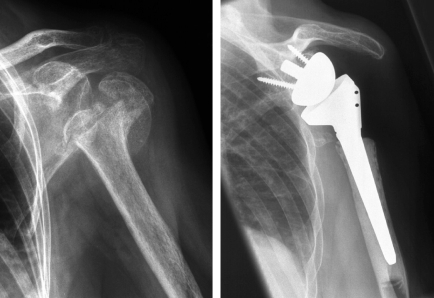
Delta III prosthesis in fracture sequelae type IV according to Boileau and Walch, with persistent dislocation and healing of the fragments with massive deformation.

20 patients had fracture sequelae (Figure 2), which were classified in 4 types according to Boileau and Walch (Boileau et al. 2006). 2 patients were type I, 2 type II, 3 type III, and 13 patients were type IV.

**Figure 3.**
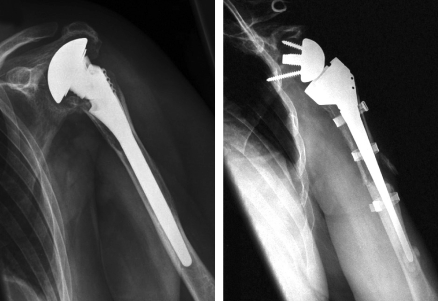
Delta III prostheses after failed fracture of prosthesis due to necrosis of the tubercles, and resulting anterior–superior migration.

Prosthesis revision was done in 16 patients (Figure 3). This was due to migration of the prosthesis due to necrosis of the tubercles in 9 cases, and secondary rotator cuff rupture in 1 case. A 2–stage prosthesis revision was necessary in 6 patients because of septic loosening. No aseptic shaft loosening, glenoid loosening, or glenoid erosions occurred in this group.

The extent of scapular notching was classified according to Sirveaux et al. (2004) using exact orthograde scans. In addition, the radiolucent lines on the glenoid were recorded according to Gonzales et al. (2006).

Rotator cuff condition and rupture size were recorded during surgery (Bateman 1963, Patte 1990).

### Statistics

Constant score values are given as mean and median values, as well as 25% and 75% percentiles.

Abduction, flexion, and strength parameters were tested non–parametrically with regard to the diagnoses using the Wilcoxon test. The p–values of each comparison were adjusted using the Bonferroni method.

Multiple regression models were calculated to compare the diagnostic significance of the Constant score with respect to diagnosis (analysis of variance with consideration of age and gender). Separate models for the follow–up examinations (pooled for months 3–36 and more) were estimated. The pooled models also included the months as covariant. The distribution of the residues was checked for normal distribution using Q–Q plots or scatter plots.

## Results

**Figure 4.**
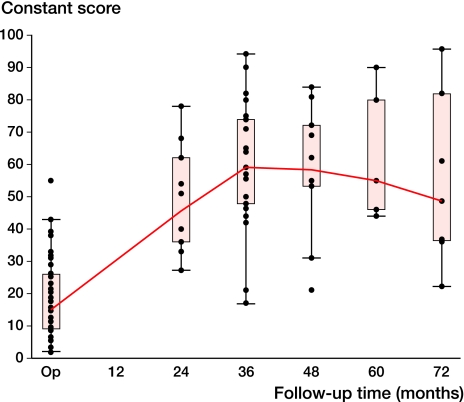
Development of the Constant score adjusted for age and gender over the period of investigation (median values, interquartile range 25% and 75%, min., max., l = outlier that lie between 1.5 and 3 times the interquartile range).

The average Constant score improved from 18 to 59 points in the overall patient population after 36 months (Figure 4), but dropped to 55 after more than 72 months.

At average follow–up, the Constant score rose from 15 (2–55) to 55 (17–96) in the overall patient population (a difference of 40), from 26 (2–55) to 74 (44–96) in CTA (a difference of 48), from 12 (2–38) to 48 (17–90) in fracture sequelae (a difference of 36), and from 10 (2–26) to 54 (21–75) points in the revision operations (a difference of 44). The global postoperative gain and the gain per diagnosis group were statistically significant (p < 0.001).

The improvement comprised all components of the Constant score, but in particular pain relief and improvement in strength (Table 1). The most pronounced relief of pain and improvement in strength were attained in the CTA group. Force could not be measured in the fracture sequelae group and the revision group preoperatively because of low abduction angles.

**Table 1.** Relief of pain and improvement in strength depending on the underlying diagnosis (mean values)

	Strength					Pain				
	max. 25 points					max. 15 points				
	Preop.	SD	Postop.	SD	p–value	Preop.	SD	Postop.	SD	p–value
Cuff tear arthropathy (n = 23)	2	4	12	6.9	< 0.001	3	4	14	2	< 0.001
Fracture sequelae (n = 20)	1	1	7	5.7	< 0.001	3	5	12	3	< 0.001
Revision arthroplasty (n = 16)	1	1	8	5.1	< 0.001	1	2	12	4	< 0.001
Total (n = 59)	1	3	9	5.9	< 0.001	2	4	13	3	< 0.001

Preop.: at baseline; Postop.: at last follow–up; CTA: cuff tear arthropathy.

These results are also reflected in the improvement in active forward flexion in the overall patient population from 47° (0–100) to 105° (30–180) (Table 2) and in active abduction from 46° (10–90) to 93° (45–180). On the other hand, external rotation with the upper arm held close to the body only increased from 9° (minus 20–5) to 19° (minus 20–80). External rotation with the arm abducted increased from –3° (–20 to 20) to 35° (0–90). It is especially striking that the improvement in external rotation in the CTA group was markedly higher (with a difference of 61) than in the fracture sequelae group (with a difference of 23) and the revision group (with a difference of 23). Preoperative and postoperative changes were statistically significant in all groups whereas differences between the groups were not (p = 0.4).

**Table 2.** Improvement in active range of motion (ROM) depending on the diagnosis. Values are mean degrees

	Total	Cuff tear arthropathy	Fracture sequelae	Revision arthroplasty
(n = 59)	(n = 23)	(n = 20)	(n = 16)
Abduction				
Preop.	46	53	30	43
Postop.	93	115	79	78
p–value	< 0.001	0.002	< 0.001	0.006
Forward flexion				
Preop.	47	52	17	53
Postop.	105	134	89	84
p–value	< 0.001	< 0.001	< 0.001	0.007
External rotation in 0° abduction				
Preop.	–9	5	–5	0
Postop.	19	28	11	12
p–value	< 0.001	< 0.001	0.01	0.02
External rotation in 90° abduction				
Preop.	–3	2	–4	–9
Postop.	35	63	19	1
p–value	< 0.001	< 0.001	0.02	0.003

Preop: at baseline; Postop: at last FU.

We observed scapular notching in 51 of 59 shoulders: Sirveaux grade 0 in 8 cases, grade 1 in 27 cases, grade 2 in 21 cases, grade 3 in 2 cases, and grade 4 in 1 case. There were no statistically significant differences between the groups. No effect on the Constant score could be seen.

Radiographically detectable limited lysis was observed once in zone 1 (upper base–plate of the glenoid component) and once in zone 2 (lower base–plate). Manifest loosening was not observed. No resorption zones were detected around the peg and the screws in the glenoid and in the humerus shaft. Heterotopic ossifications occurred in the region of the lower joint capsule in 14 cases. However, a bony spur was present on the medial margin of the scapular notch in 38 patients (≤ 2 mm in 17, ≤ 4 mm in 11, ≤ 6 mm in 5, and ≥ 6 mm in 5). Consequently, the notching appeared deeper than it actually was.

We noted 3 cases of dislocation (in 1 instance because of trauma/fall), 1 case of acromion fracture, 1 case of fracture of the coracoid process, and 2 disconnections of the shaft components associated with massive scapular notching. Hematomas led to revisions in 5 cases. Infections were observed in 5 joints. All infections occurred after prior surgery, i.e. 3 after osteosynthesis and 2 after prosthesis revision because of deep infection. This means that 2 of the 6 patients who were admitted to the hospital with a pre–existing prosthesis infection had a reinfection. Overall revisions were necessary in 15 patients. Transient neurological deficits were observed in 2 patients.

## Discussion

Implantation of a reversed prosthesis restores a stable center of rotation and thus provides the prerequisites for restoration of function. Considering the often catastrophic initial situation, the outcome was surprisingly good. It is not so much the plain postoperative functional score that is important but rather the gain in function, as a measure for comparison of the preoperative and postoperative state (the “Delta constant”). In the individual patient, a low postoperative value in absolute terms may nevertheless signify a major improvement compared to the preoperative situation.

The Constant score obtained in our study was lower than or similar to that in the literature (Table 3). However, the initial preoperative values were also lower in all 3 groups. In view of the low initial values, it is on a level similar to that for data reported in the literature. The greatest improvement in Constant score was achieved in CTA group, followed by revision arthroplasty and fracture sequelae. This is to some extent contrary to the results published in the literature. In most papers, the fracture sequelae attained a higher level than revisions (Boileau et al. 2006a, Jouve et al. 2006, Wall et al. 2007). We attribute the somewhat smaller functional gain in the CTA group in our study to the strict selection criteria used, i.e. the fact that we only included patients with advanced CTA. By contrast, in the literature reversed prostheses are also recommended for complete rotator cuff ruptures in the absence of obvious degenerative joint lesions (Gonzales et al. 2006, Molé et al. 2006). We prefer to perform a muscle transfer operation (latissimus dorsi or pectoralis major) in cases of irreparable rotator cuff defect without joint deterioration—even in the case of a failed cuff repair—as opposed to a reversed prosthesis (Irlenbusch et al. 2003, 2008a, b, Gerber et al. 2006). For this reason, our CTA group comprised exclusively patients with advanced cuff tear arthritis, especially of Hamada grade 4 and 5 (Hamada et al. 1990).

**Table 3.** Published results on reversed shoulder replacement

A	B	C	D	E		F		G		H		I	
Sirveaux 2004	CTA	80	44	73	138	4 ^c^	11 ^c^	23 P	66 P	–	–	2.7 ^b^	13.4 ^b^
						17 ^d^	40 ^d^						
Frankle 2005	CTA	60	36	55	105	12	41	–	–	34	68	6.3 ^a^	2.2 ^a^
Seebauer 2005	CTA	57	18	–	–	–	–	26–43%	85–97%	–	–	–	12.8 ^b^
Boileau 2006	Diff.	45	40	55	121	7	11	17 P	58 P	–	–	–	3.2 ^a^
Favard 2006	CTA	127	49	70	135	8 ^c^	23 P	65 P	–	–	3 ^b^	13 ^b^	46 ^d^
Gohlke 2006	Revision fracture prostheses	20	18	–	–	–	–	18%	63%	–	–	88%	good or excellent
Gonzales 2006	CTA, revision	42	50	82	123	5	7	25 P	56 P	–	–	3.3 ^b^	11.1 ^b^
Jouve 2006	Diff.	65	46	49	116	2 ^c^	2.5 ^c^	17 P	49 P	–	–	3.2 ^b^	10.5 ^b^
						12 ^d^	27 ^d^						
Klein 2006	Diff.	46	13	–	–	–	–	–	86%	–	–	–	–
Molé 2006	RC rupture	47	30	80	133	10	13	40%	90%	–	–	4 ^b^	12.7 ^b^
Paladini 2005	Revision head prostheses	7	36	–	–	–	–	23 P	49 P	–	–	–	–
Wall 2006	Revision total arthroplasty	24	38	61	114	18 ^c^	11 ^c^						
						51 d	46 d	33%	71%	–	–	5.3 ^b^	11.1 ^b^
Werner/Gerber 2006	CTA	58	38	42	100	17 ^c^	12 ^c^	29%	64%	–	–	5.2 ^b^	10.5 ^b^
Irlenbusch 2009	Diff.	59	46	47	105	–3 ^d^	35 ^d^	15 P	55 P	–	–	2.6 ^b^	12.9 ^b^

^a^ max. 10 points/VAS.

^b^ max. 15 points.

^c^ 0° abduction.

^d^ 90° abduction.

A Authors B Diagnosis CTA: cuff tear arthropathy; RC rupture: rotator cuff rupture; Diff.: different diagnosis groups; C n D Follow–up (months) E Forward flexion (°), pre/post F External rotation (°), pre/post G Constant score, pre/post P: points in Constant score; %: corrected Constant score. H ASES, pre/post I Pain, pre/post

Functional improvement is particularly attributable to the increase in flexion and abduction, but not to external rotation, which did not usually improve—or not significantly. Thus, various authors now evaluate the degree of active external rotation (specifically of the teres minor muscle) preoperatively using MRI and, if appropriate, carry out a latissimus dorsi transfer in the same session (Gerber et al. 2007, Boileau et al. 2008). Restoration of useful external rotation is possible in some patients, even without any muscle transfer operation. This depends in particular on the presence of a functional infraspinatus and teres minor (Simovitch et al. 2007a). In our patients, it was striking that the external rotation results achieved in the CTA group were markedly better than those in the fracture sequelae group and in the prosthesis revision group (Table 2). This indicates that care should be taken, if anatomically possible, to ensure meticulous reconstruction of the rotator cuff, even in reversed prosthesis implantation. It was not possible to appraise the significance of the condition of the teres minor muscle in our study since MRI films were not available in all patients preoperatively.

Looking at the individual components of the Constant score, pain relief and increase in strength appear to be the most important parameters affecting the general improvement of function (Table 1). This is, of course, mainly due to the number of points attributed to them in the Constant evaluation system (15 and 25 points, respectively) as compared to a maximum of 10 points for the other parameters. It is noteworthy that once again our best results were attained in the CTA group, whereas the fracture sequelae group and the prosthesis revision group showed a lower increase in strength and a lower degree of pain relief. This is consistent with the data published by Wall et al. (2007).

In our opinion, the most interesting result of our investigation was the continuous fall in score values starting after 3 years postoperatively (Figure 4). Several factors would probably explain this decline. Less intensive physiotherapy may play a major role, and patients adapt to an increasing loss of function.

Several factors contributed to the low dislocation rate (3/59) in our series compared to up to 25% in the literature. Important factors are meticulous positioning of the implant components, pre–tensioning of the deltoid and coracobrachial muscles, extensive mobilization, and resection of scarred and contracted parts of the capsule and bone fragments, as well as the best possible reconstruction of the external and internal rotators of the rotator cuff (Boileau et al. 2006a, b, Favard et al. 2006, Wall et al. 2006).

In a survey of the literature, we found relatively high levels of scapular notching (Sirveaux et al. 2004, Werner et al. 2005, Simovitch et al. 2007b). The low rate of dislocation and revision indicates that mistakes in the surgical technique cannot account for the high incidence of scapular notching. Even mild degrees of osteolysis (Sirveaux I and II) are detected using a meticulous radiographic technique. It must be borne in mind that the confirmation of a notching of grade 1 or 2 according to Sirveaux requires an exactly orthograde scan. Otherwise, an incorrectly low notching rate is registered (Sirveaux et al. 2004). It must also be considered that it was not common practice to implant the metal back (metaglene) in a caudal position, consequently at the time of implantation, as recommended today (Nyffeler et al. 2005).

The revision rate (15/59) is similar to that reported in the literature (17–50%) (Werner et al. 2005, Frankle et al. 2006, Molé et al. 2006, Wall et al. 2006, Levy et al. 2007). All revisions were performed in patients with fracture sequelae or prosthesis revision. Similarly, a lower rate of reoperations has been reported in the literature for CTA patients than for those with fracture sequelae or prosthesis revision (Boileau et al. 2006b, Wall et al. 2007). Also, all cases requiring revision due to infection involved patients with preoperative fracture sequelae or revision of a prosthesis.

We found a complication rate of one–third when scapular notching was not taken into account. This is similar to that reported by Levy et al. (2007) for prosthesis revision. Counting all complications (including minor ones), Wall et al. (2006) and Werner et al. (2005) found a complication rate as high as 50%.

In agreement with numerous reports in the literature, our findings indicate that the reversed shoulder prosthesis must be regarded as a salvage procedure despite the astonishingly good results. The gain of function is impressive and depends strongly on etiology, but there are high rates of complications and revisions.
